# Recent Status of Laparoscopic Distal Gastrectomy in Korea: A Multicenter Retrospective Cohort Study (Pre-study Survey of KLASS-07 Trial)

**DOI:** 10.3389/fonc.2019.00982

**Published:** 2019-10-03

**Authors:** Chang In Choi, Chang Min Lee, Ji Ho Park, Ye Seob Jee, Han Hong Lee, Oh Jeong, Sungsoo Park

**Affiliations:** ^1^Department of Surgery, Biomedical Research Institute, Pusan National University College of Medicine, Busan, South Korea; ^2^Department of Surgery, Korea University College of Medicine, Seoul, South Korea; ^3^Department of Surgery, Gyeongsang National University School of Medicine, Jinju, South Korea; ^4^Department of Surgery, Dankook University College of Medicine, Cheonan, South Korea; ^5^Department of Surgery, College of Medicine, The Catholic University of Korea, Seoul, South Korea; ^6^Department of Surgery, Chonnam National University Medical School, Gwangju, South Korea; ^7^Department of Surgery, Korea University Medical Center Anam Hospital, Seoul, South Korea

**Keywords:** gastric cancer, gastrectomy, laparoscopic surgery, baseline survey, multicenter study

## Abstract

**Purpose:** To analyze the surgical trend and brief postoperative results of laparoscopic distal gastrectomy (LDG) in Korea on the basis of a multicenter cohort.

**Materials and Methods:** Data of 812 patients who underwent LDG between January and December 2016 were collected from 14 surgeons at 7 institutions. Patients were divided into laparoscopy-assisted distal gastrectomy (LADG) group and totally laparoscopic distal gastrectomy (TLDG) group. Perioperative and clinicopathologic outcomes were compared retrospectively.

**Results:** Among the patients [*n* = 222 (27.3%) LADG; *n* = 590 (72.7%) TLDG], there are no significant differences in patient's demographics (sex, age, body mass index, and American Society of Anesthesiologists score). Billroth-I anastomosis (84.7%) was most performed in the LADG group, but Billroth-II anastomosis (59.0%) in the TLDG group (*p* < 0.001). The mean operative time was longer in the TLDG group (197.3 ± 44.4 min vs. 222.0 ± 60.2 min, *p* < 0.001), and there was no statistical difference in the hospital stay between the two groups (9.6 ± 4.8 days vs. 8.9 ± 7.1 days, *p* = 0.149). There were no significant differences in morbidity and mortality between the two groups. The length of proximal margin was longer in the TLDG group (4.3 ± 3.1 cm vs. 6.0 ± 3.4 cm, *p* < 0.001), but the distal margin was longer in the LADG group (6.5 ± 3.7 cm vs. 5.5 ± 3.1 cm, *p* < 0.001). The distribution of operations among each institution was shown very heterogeneously.

**Conclusion:** There was no significant difference related to surgical outcome between LADG and TLDG in pre-study survey prior to KLASS-07 trial. Therefore, to obtain more reliable data, well designed prospective randomized controlled study is needed.

## Introduction

Since the first report of laparoscopic gastrectomy for gastric cancer by Kitano et al. minimally invasive surgery has been developed steadily in the recent two decades. Laparoscopic surgery has various advantages such as less postoperative pain, inflammatory response, rapid recovery, early discharge, and excellent cosmetic result compared with conventional open laparotomy (1–4). Several studies have demonstrated that the oncologic safety of laparoscopic gastrectomy is similar to conventional open gastrectomy for early gastric cancer (5, 6), and laparoscopic gastrectomy was already accepted as the standard treatment option for early gastric cancer as well as benign gastric tumor. In addition, laparoscopic gastrectomy widens its boundary with the development of surgical skill and instruments even to locally advanced gastric cancer on the basis of evidence from several retrospective studies (7–9). With this, large-scale prospective studies are ongoing, and the final results are being awaited (KLASS-02, JLSSG0901) (10, 11).

Laparoscopy-assisted gastrectomy required a mini-laparotomy on the epigastrium for gastric division and anastomosis after laparoscopic gastric mobilization (extracorporeal anastomosis). In cases that a patient is obese or has short duodenum, the anastomosis could not be easy under the narrow working space of the mini-incision, whereas in the totally laparoscopic gastrectomy, the whole procedure from the gastric division including lymphadenectomy to the anastomosis is performed intracorporeally. It has various advantages, such as the superiority of the cosmetic result due to umbilical incision and convenient anastomosis under good operative view even in obese patients. According to accumulated laparoscopic surgical experience, the recent surgical trend shifted from laparoscopy-assisted gastrectomy to totally laparoscopic gastrectomy (12).

However, a prospective randomized controlled study (RCT) comparing the postoperative outcome and patient's life of quality (QoL) is still rare, although there were several retrospective studies between these two procedures. Thus, authors are preparing for a multicenter prospective study comparing the QoL and postoperative outcome between laparoscopy-assisted distal gastrectomy (LADG) and totally laparoscopic distal gastrectomy (TLDG) and conducted a brief survey regarding the surgical trend among Korean gastric surgeons as reference for a subsequent study (KLASS-07 trial). Therefore, this study aimed to analyze the current status and surgical trend of laparoscopic distal gastrectomy in Korea.

## Methods

This study was designed as a multicenter retrospective cohort study. Medical data of 812 patients were collected retrospectively using the same case report form provided by 14 gastric surgeons of seven institutions, which are affiliated to the KLASS-07 trial organizing committee. Between January and December 2016, patients who were diagnosed with gastric adenocarcinoma or neuroendocrine carcinoma underwent laparoscopic distal gastrectomy. All patients were compared by dividing them into two groups: LADG and TLDG group. Pylorus-preserving gastrectomy (PPG) was excluded in this database as it is not a distal gastrectomy despite a partial gastrectomy.

Patients' demographics, postoperative outcome, and pathologic data were analyzed, and all continuous data are expressed as mean ± standard deviation. Categorial variables were assessed by Pearson's chi-square test and Fisher's exact test, and continuous variables were assessed by Student's *t*-test. American Society of Anesthesiologists (ASA) scores between the two groups were compared (1 vs. others). We described all anastomosis methods separately. However, in the statistical analysis, we included Billroth II with Braun anastomosis (B-IIb) to Billroth II anastomosis (B-II), and uncut Roux-en-Y anastomosis (REY) to Roux-en-Y anastomosis to reduce the errors. In a comparison of the resectability, complete resection case was compared with incomplete resection case (R1 and R2). Moreover, in the analysis of the World Health Organization classification, most common tubular adenocarcinoma was compared with signet ring cell carcinoma because the pathologic entities of other gastric cancers were very rare. Subgroup analysis was performed for the operation time according to the reconstruction method in each group. For this, one-way analysis of variance and Bonferroni *post-hoc* analysis were used. In the LADG group, uncut REY anastomosis was included in REY anastomosis for the statistical calculation.

To visualize patients' distribution according to each institution, jittered scatterplot was applied using the following formula: (measured value) + (R-0.5) X 0.3, where “R” is a random number from zero to one. For all analyses, *p* < 0.05 was considered significant statistically and SPSS version 22.0 for Windows (SPSS, Inc., Chicago, IL) was used for statistical analysis. This study was reviewed and approved by the Institutional Review Board of Pusan National University Hospital (H-1803-023-064). And informed written consent in terms of using their medical records was provided to all patients and their legal guardian before study enrollment.

## Results

### Patient Demographics and Perioperative Data

A total of 222 (27.3%) patients underwent LADG and 590 (72.7%) patients underwent TLDG. Of the 812 patients, 511 (62.9%) were men, with a mean age of 61.9 ± 11.4 years and mean body mass index (BMI) of 24.6 ± 13.4 kg/m2. There were no significant differences in sex, age, BMI, and ASA scores between the two groups (Table 1).

**Table 1:** Patients' demographics.

**Variables**	**LADG** **(*n* = 222)**	**TLDG** **(*n* = 590)**	**Overall** **(*n* = 812)**	***p*-value**
Sex				0.569
Male	136 (61.3)	375 (63.6)	511 (62.9)	
Female	86 (38.7)	215 (36.4)	301 (37.1)	
Age (years)	61.5 ± 10.3	62.0 ± 11.8	61.9 ± 11.4	0.558
BMI (kg/m^2^)	23.9 ± 3.0	24.8 ± 15.6	24.6 ± 13.4	0.367
ASA score				0.637^a^
1	52 (23.5)	129 (22.0)	184 (22.4)	
2	165 (74.7)	406 (69.2)	571 (70.7)	
3	4 (1.8)	50 (8.5)	54 (6.7)	
4	0	2 (0.3)	2 (0.2)	

*LADG, laparoscopy-assisted distal gastrectomy; TLDG, totally laparoscopic distal gastrectomy; ASA, American Society of Anesthesiologists*.

a *ASA score 1 vs. Others*.

Postoperative data are presented in Table 2. In all patients, B-II was most performed in 357 (44.0%) patients and Billroth I anastomosis (B-I) was performed in 213 (26.1%) patients. With regard to the anastomosis method, B-I was performed in 188 (84.7%) patients in the LADG group and 24 (4.1%) patients in the TLDG group. B-II was performed in 9 (4.1%) patients in the LADG group and 348 (59.0%) patients in the TLDG group. Moreover, REY was performed in 18 (8.1%) patients in the LADG group and in 114 (19.4%) patients in the TLDG group. There was a significant difference in the anastomosis method between the two groups (*p* < 0.001). Both groups were mostly anastomosed with the stapling method.

**Table 2:** Postoperative data.

**Variables**	**LADG** **(*n* = 222)**	**TLDG** **(*n* = 590)**	**Overall** **(*n* = 812)**	***p*-value**
Reconstruction method				<0.001^a^
Billoth-I	188 (84.7)	24 (4.1)	212 (26.1)	
Billoth-II	9 (4.1)	348 (59.0)	357 (44.0)	
Billoth-II + Braun	6 (2.7)	50 (8.5)	56 (6.9)	
Uncut Roux-en-Y	1 (0.5)	54 (9.2)	55 (6.8)	
Roux-en-Y	18 (8.1)	114 (19.3)	132 (16.3)	
Reconstruction manner				1.000
Stapling	222 (100.0)	589 (99.8)	811 (99.9)	
Manual	0	1 (0.2)	1 (0.1)	
LND extent				<0.001
D1	2 (0.9)	17 (2.9)	19 (2.3)	
D1+	40 (18.0)	466 (79.0)	506 (62.3)	
≥D2	180 (81.1)	107 (18.1)	287 (35.3)	
Co-resection				0.009
Yes	33 (14.9)	49 (8.3)	82 (10.1)	
No	189 (85.1)	541 (91.7)	730 (89.9)	
Curability				0.199^b^
R0	222 (100.0)	583 (98.8)	805 (99.1)	
R1	0	4 (0.7)	4 (0.5)	
R2	0	3 (0.5)	3 (0.4)	
Operative time (min)	197.3 ± 44.4	222.0 ± 60.2	215.2 ± 57.4	<0.001
Hospital stay (days)	9.6 ± 4.8	8.9 ± 7.1	9.1 ± 6.6	0.149
Morbidity^c^	18 (10.6)	63 (11.8)	81 (11.5)	0.783
Mortality	1 (0.5)	2 (0.3)	3 (0.4)	1.000

*LADG, laparoscopic assisted distal gastrectomy; TLDG, totally laparoscopic distal gastrectomy; DG, distal gastrectomy; LND, lymph node dissection*.

a *B-I vs. B-II (+Braun) vs. Roux-en-Y (+Uncut)*.

b *R0 vs. R1 and R2*.

c *There were 106 missing values of total 812 cases*.

D2 lymphadenectomy (180 patients, 81.1%) in the LADG group and D1+ lymphadenectomy in the TLDG groups (466 patients, 79.0%) were mostly performed (*p* < 0.001). Frequency of co-resection was higher in the LADG group than in the TLDG (14.9% vs. 8.3%, *p* = 0.009). The mean operative time was longer in the TLDG group than in the LADG group (197.3 ± 44.4 min vs. 222.0 ± 60.2 min, *p* < 0.001). Morbidity was slightly higher in the TLDG group; however, it was not significant statistically (18 patients, 10.6% vs. 63 patients, 11.8%, *p* = 0.783). One patient died (0.5%) in the LADG and two (0.3%) in the TLDG, and no difference was found between the two groups.

### Pathological Data

Overall, the mean tumor size was 3.0 ± 2.1 cm, and tumor size was larger in the LADG group, but no difference was shown between the two groups (3.3 ± 2.1 cm vs. 2.9 ± 2.1 cm, *p* = 0.058). In the LADG group, the tumor located in mid-part of the stomach was 115 (51.8%) patients and in the lower part was 95 (42.8%) patients, whereas the middle tumor was 36.9% and the lower tumor was 62.5% in the TLDG group (*p* < 0.001). Moderately differentiated tubular adenocarcinoma (tub MD) was the most common in 239 (29.4%) of all patients, poorly differentiated tubular adenocarcinoma (tub PD) was confirmed in 218 (26.5%) patients, and cohesive carcinoma (SRC) was identified in 172 (21.2%) patients. In the LADG group, tub MD and SRC were diagnosed finally in each 70 (31.5%) patient and 66 (29.7%). In the TLDG group, tub MD and tub PD were confirmed in 169 (28.6%) and 163 (27.6%) patients, respectively. In Lauren's classification, the incidence of intestinal and diffuse type was comparable (45.4% vs. 46.3%) in the LADG group. In the TLDG group, the intestinal type was greater than the diffuse type (59.0% vs. 23.8%), and there was a significant difference in the final pathologic finding (p < 0.001). The retrieved lymph node was significantly greater in the TLDG group (42.6 vs. 46.3, *p* = 0.008), and there were no differences in metastatic lymph nodes between the two groups. Resection margin showed significant differences in both groups. The length of the proximal margin (PRM) was longer in the TLDG group (4.3 ± 3.1 cm vs. 6.0 ± 3.4cm, *p* < 0.001) and distal margin (DRM) was longer in the LADG group (6.5 ± 3.7 cm vs. 5.5 ± 3.1 cm, *p* < 0.001). All pathologic data are presented in Table 3.

**Table 3:** Pathological data.

**Variables**	**LADG** **(*n* = 222)**	**TLDG** **(*n* = 590)**	**Overall** **(*n* = 812)**	***p*-value**
Tumor size (cm)	3.3 ± 2.1	2.9 ± 2.1	3.1 ± 2.1	0.058
Tumor location				<0.001
Upper	12 (5.4)	3 (0.5)	15 (1.8)	
Middle	115 (51.8)	218 (36.9)	333 (41.0)	
Lower	95 (42.8)	369 (62.5)	464 (57.1)	
WHO classification				<0.001^a^
Papillary	3 (1.4)	2 (0.3)	5 (0.6)	
Tub WD	29 (13.1)	128 (21.7)	157 (19.3)	
Tub MD	70 (31.5)	169 (28.6)	239 (29.4)	
Tub PD	52 (23.4)	163 (27.6)	215 (26.5)	
Mucinous	0	9 (1.5)	9 (1.1)	
Cohesive (SRC)	66 (29.7)	106 (18.0)	172 (21.2)	
Others	1 (0.5)	10 (1.7)	11 (1.4)	
Unknown	1 (0.5)	3 (0.5)	4 (0.5)	
Lauren				<0.001
Intestinal	99 (45.4)	329 (59.0)	428 (55.2)	
Diffuse	101 (46.3)	133 (23.8)	234 (30.2)	
Mixed	181 (8.3)	96 (17.2)	114 (14.7)	
Retrived lymph nodes	42.6 ± 15.9	46.3 ± 17.9	45.3 ± 17.5	0.008
Metastatic lymph nodes	0.5 ± 2.1	0.9 ± 3.6	0.8 ± 3.3	0.149
T stage				0.002
T1a	115 (52.0)	273 (46.7)	388 (48.1)	
T1b	87 (39.4)	193 (33.0)	282 (35.0)	
T2	10 (4.5)	58 (9.9)	67 (8.3)	
T3	2 (0.9)	33 (5.6)	34 (4.2)	
T4a	7 (3.2)	28 (4.8)	35 (4.3)	
N stage				0.267
N0	193 (86.9)	492 (83.4)	685 (84.4)	
N1	16 (7.2)	54 (9.2)	70 (8.6)	
N2	9 (4.1)	19 (3.2)	28 (3.4)	
N3	4 (1.8)	25 (4.2)	29 (3.6)	
TNM stage^b^				0.031^c^
IA	182 (82.4)	430 (73.5)	612 (75.9)	
IB	18 (8.1)	65 (11.1)	83 (10.3)	
IIA	9 (4.1)	32 (5.5)	41 (5.1)	
IIB	8 (3.6)	23 (3.9)	31 (3.8)	
IIIA	1 (0.5)	10 (1.7)	11 (1.4)	
IIIB	2 (0.9)	10 (1.7)	12 (1.5)	
IIIC	1 (0.5)	15 (2.6)	16 (2.0)	
Proximal margin (cm)	4.3 ± 3.1	6.0 ± 3.4	5.5 ± 3.4	<0.001
Distal margin (cm)	6.5 ± 3.7	5.5 ± 3.1	5.8 ± 3.3	<0.001
ESD before surgery	18 (8.1)	29 (4.9)	47 (5.8)	0.092

*LADG, laparoscopy-assisted distal gastrectomy; TLDG, totally laparoscopic distal gastrectomy; Tub, tubular adenocarcinoma; WD, well-differentiated; MD, moderate-differentiated; PD, poorly-differentiated; SRC, signet-ring cell; ESD, endoscopic submucosal dissection. Some missing values were excluded in a calculation*.

a *Tub vs. SRC*.

b *TNM stage was analyzed with AJCC 7th edition*.

c *Stage I vs. II vs. III*.

### Distribution of Patients According to the Institution

**Figure 1:**
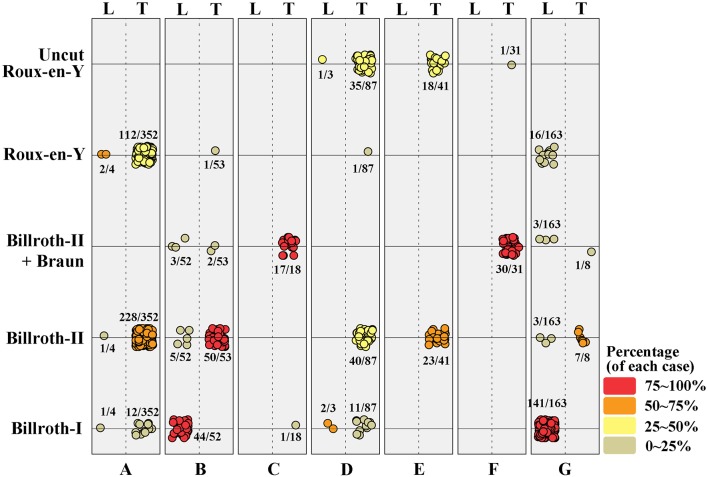
Distribution of surgical procedures in each institution. The value was colored and visualized according to its percentage. L, laparoscopy-assisted distal gastrectomy; T, totally laparoscopic distal gastrectomy.

Collected patients' data by each institution shows very heterogeneous distribution, and it was difficult to find any regularity. B-I was performed in 212 patients, of which 24 (4.1%) underwent intracorporeal B-I (delta anastomosis) in the TLDG group and 188 (84.7%) patients underwent extracorporeal B-I in the LADG group. B-II was performed in 357 patients, but it was performed in only 9 (4.1%) patients through LADG and most patients underwent intracorporeal B-II through TLDG. B-IIb, REY, and uncut REY were performed in 56, 132, and 55 patients, respectively. This may show various results according to the policy of the institutions. laparoscopy-assistedDetails of the patient distribution are visualized in Figure 1.

### Comparison of the Operation Time According to the Reconstruction Methods in Each Group

**Figure 2:**
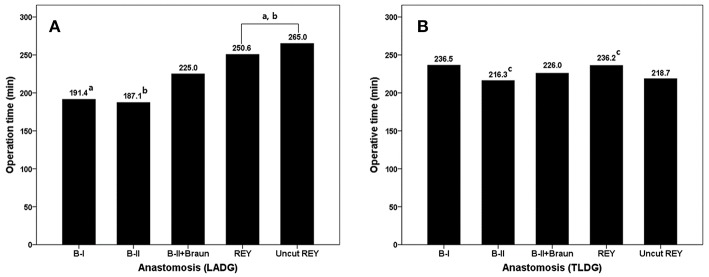
Comparison of the operation time according to the reconstruction methods in each group. Bonferroni *post-hoc* analysis was used and statistical different values were marked lowercase a, b, and c.

In the LADG group, the overall operation time of B-I and B-II reconstruction is relatively shorter than others (191.4 and 187.1 min). B-I and B-II groups in LADG showed significant differences compared with REY group (including uncut REY, *p* < 0.001 and 0.001). In the TLDG group, there were no big numerical differences in the operation time among each reconstruction methods. The overall operation time was longest in B-I reconstruction (delta anastomosis) group (236.5 min). And there was a significant difference between B-II and REY group in Bonferroni *post-hoc* analysis (216.3 min vs. 236.2 min, *p* = 0.022). The statistical difference was presented as the lowercase a, b, and c in Figure 2.

## Discussions

Laparoscopy-assisted gastrectomy has been used for a long time with increasing popularity from an era of early laparoscopic surgery until the present. In this procedure, gastric mobilization and lymph node dissection are carried out laparoscopically, and the anastomosis is performed extracorporeally through the mini-laparotomy in the epigastrium. However, anastomosis of intestines in very obese patients or patients with thick abdominal wall could be difficult because excessive traction is needed and the operative visual field is poor in narrow and restricted space of the upper abdominal cavity. Totally laparoscopic gastrectomy, in which the whole operation is carried out intracorporeally, enables anastomosis of intestine more safely and conveniently, as the anastomosis site can be monitored directly under the laparoscopic view.

The term totally laparoscopic gastrectomy was first used in 1999 by Mayers in his report of intracorporeal B-I anastomosis (13). Thereafter, intracorporeal anastomoses using various methods have been reported, and the recent surgical trend has progressed to totally laparoscopic gastrectomy with high interest in minimally invasive surgery. Ikeda et al. compared LADG with TLDG for 80 gastric cancer patients, and they reported no significant difference in operation time, harvested lymph node, and morbidity; however, TLDG showed less blood loss and rapid recovery compared with LADG (12). Kim et al. compared the postoperative outcome related to BMI between LADG and TLDG. They reported that there was no difference in major complication in an obese patient with BMI more than 25 kg/m2 between two groups, but in the LADG group, the overall complication was higher, and recovery after surgery (such as dietary progression, first flatus, and hospital stay) was slower than that in the TLDG group (14). Kanaji also anticipated that TLDG with a short hospital stay, wide working space, and small wound size could replace LADG via a prospective randomized controlled study (15). Han et al. suggested that TLDG is superior to LADG in terms of operative time, blood loss, hospital stay, and cosmetic result (16). Lee et al. reported that the inflammatory response might be lower in TLDG by less tissue damage because it does not require excessive traction of the stomach through the mini-laparotomy for anastomosis (17). Recently, Lin et al. suggested that the number of harvested lymph node was higher in TLDG, but there were no differences in other factors related to postoperative outcome and recovery (18). Similarly, numerous studies compared TDLG with LADG. However, those were mostly single-sectional retrospective study with inconsistent and varied results.

In a recent meta-analysis by Zhang et al. there were no differences in the operative time, analgesic use, first flatus, and overall complication between LADG and TLDG, but TLDG was superior to LADG in terms blood loss, number of harvested lymph node, and hospital stay (19). However, high-level evidences are difficult to obtain through meta-analysis because of the rarity of prospective RCTs for TLDG. Through their prospective RCT for 110 gastric cancer patients in 2015, Woo et al. reported that early surgical outcome (including the complication) and QoL did not show differences between LADG and TLDG. This was only a single-institution trial, but a markedly valuable study. As mentioned above, there have been some papers comparing the TDLG and LADG. And many authors have emphasized the feasibility or superiority of the TLDG. However, the LADG is still performed in some institutions although recent surgical trend moves to the TLDG. To obtain more reliable data for the postoperative outcome (including quality of life), a well-designed multicenter prospective RCT is needed.

The KLASS-07 trial is a multicenter prospective RCT which compares the QoL of patients who underwent LADG and TLDG. At this time, recruiting researcher was closed with support of the Korean Laparoscopic Gastrointestinal Surgery Study Group and a review of the institutional review board for the study is in progress. This brief survey of the current status of domestic gastric cancer surgery was performed as reference for the study protocol. Of the total 891 patients, 591 (71.4%) underwent TLDG, and there were no significant differences in patients' demographics. This reflects that the recent trend of the laparoscopic gastrectomy shifted from LADG to TLDG. laparoscopy-assisted.

In LADG, B-I was the most common anastomosis (84.7%). In the TLDG group, B-I (delta anastomosis) was only 4.1%, B-II was 59.0%, and REY was 19.3%. Delta anastomosis was first introduced by Kanaya et al. in 2002, and many later studies concluded that it was a safe and feasible procedure clinically (20). However, our result implies that the delta anastomosis has still many difficulties to be accepted as the standard anastomosis technique for TLDG.

The co-resection was higher in the LADG group than in the TLDG group, but most cases were cholecystectomy and it may not have clinical significance. The operation time was longer in the TLDG because intracorporeal gastrojejunostomy, such as B-II or REY anastomosis, is a time-consuming procedure. The hospital stay in the TLDG group was shorter, but it was not statistically significant, and there were no differences in the morbidity and mortality between the two groups. This result is consistent with other studies.

The number of harvested lymph node was significantly higher in the TLDG group. However, lymph node dissection during the LADG and TLDG is performed in the same manner. Because more than 40 lymph nodes were resected in both groups, it could be not a factor affecting the clinical course. In a comparison of TNM stage, T1, N0, and stage I were most frequent for all patients. This could mean that many surgeons are still selecting patients for laparoscopic approach for gastric cancer. This may be due to the lack of results regarding the safety and long-term outcome of laparoscopic gastrectomy for advanced gastric cancer (KLASS-02, JLSSG0901). Moreover, in this study, there were more early cancer cases in the LADG group, and we think that has affected the result from the institutional policy for the indication of laparoscopic gastrectomy.

In the TLDG group, PRM was longer and DRM was shorter than that in the LADG group. This may be associated to the tumor location of the TLDG group, which was lower than that in the LADG group, and lesion localization is known to be difficult in the intracorporeal anastomosis during TLDG because surgeons cannot manually palpate the lesion directly. Therefore, the surgeon's concern about obtaining clear margin leads to wider resection, and preference to gastrojejunostomy rather than gastroduodenostomy during TLDG could also influence the result (16, 21). However, Shinohara et al. suggested no significant differences in the length of PRM between the two groups, and Jeong et al. reported that PRM was rather shorter in the TLDG group (22, 23). In these studies, gastroduodenostomy (B-I, delta anastomosis) was mostly performed after TLDG. We speculated that the results could be affected by the difference in the anastomosis method.

The patient distribution according to participant institution is visualized in Figure 1, which indicates that five of the seven institutions preferred TLDG and one toward LADG. In addition, one institution which performed LADG mostly chose gastroduodenostomy as the standard anastomosis procedure, it may cause a deviation of the extracorporeal B-I anastomosis in the LADG group. REY is known to increase gradually in Korea; however, B-II is more commonly used than REY as an alternative method to intracorporeal gastroduodenostomy (delta anastomosis) after TLDG, and uncut REY or B-IIb is also performed in some cases. Although super high-volume centers, with around 1,000 gastrectomies performed, were excluded in this study, we think that the heterogeneity of the results in this study might reflect the current status of the gastric surgeon's society in Korea, because the anastomosis method after the TLDG was not standardized among institutions and surgeons. These points emphasize the necessity of a multicenter RCT for comparing TLDG with LADG. While many previous studies have focused on the postoperative outcomes, this study shows an aspect of the recent laparoscopic gastrectomy including the perioperative data between two groups. It can be one of the strengths of the multicenter cohort study.

There are some differences in the operation time according to the anastomosis methods in each group. Overall operation time was higher in the TLDG group than the LADG group. This may cause to take more time for tumor localization and anastomosis in TLDG. Although we couldn't evaluate pure anastomosis time in each group, LADG with B-I and B-II showed relatively short operation time. We think that it is a reasonable result because Braun anastomosis and Roux-en-Y need additional jejunojejunostomy. Whereas, TLDG with intracorporeal B-I (delta) anastomosis showed the longest operation time. Delta anastomosis uses more stapler compared with extracorporeal B-I anastomosis, however, it has been known as not time-consuming procedure. Finally, delta anastomosis might be the unfamiliar or not preferred method to participants in this study. This deviation between extra- and intracorporeal B-I anastomosis became the background to exclude the B-I anastomosis.

This preliminary study has several limitations. First, the number of 812 cases is relatively small to represent the surgical trend in Korea, even if it was not a small cohort. However, it could be significant data as they were from various institutions. Second, as mentioned, the anastomosis methods are very heterogeneous among institutions. However, it will be thoroughly controlled by the study protocol in a subsequent prospective RCT (KLASS-07), from which more reasonable results could be obtained for TLDG and LADG. Consequently, gastroduodenostomy was finally excluded from KLASS-07 trial protocol due to selection deviation between two groups. Third, there were 107 missing values among the 812 patients in the analysis of postoperative complications. Moreover, the collected complication data were not classified according to severity grade, such as Clavien-Dindo classification. However, the overall complication rate in this study was around 10%, and we believe that this could be an acceptable result comparing other studies. Because the surgical technique and postoperative management have been shared among surgeons within the surgical society, the morbidity rate of missed values would not show a big difference in the collected data.

## Conclusion

This is a preliminary study conducted before starting the KLASS-07 trial and our data shows there were no significant differences in postoperative results between LADG and TLDG. Many surgeons still perform the laparoscopic gastrectomy using various techniques according to their own policy because there is no strong consensus statement related to LADG and TLDG. Although this study can hardly represent the surgical trend of Korean gastric surgeons, it might be a meaningful reference for a multicenter trial.

## Data Availability Statement

The datasets generated for this study are available on request to the corresponding author.

## Author Contributions

CC performed the statistical analysis, prepared the manuscript, and drafted this manuscript. SP supervised and organized the study. CL, JP, YJ, HL, and OJ contributed to manuscript modification. All authors contributed to data acquisition and read and approved the final manuscript.

### Conflict of Interest

The authors declare that the research was conducted in the absence of any commercial or financial relationships that could be construed as a potential conflict of interest.
